# Novel nanosuspension‐based dissolving microneedle arrays for transdermal delivery of a hydrophobic drug

**DOI:** 10.1002/jin2.41

**Published:** 2018-06-27

**Authors:** Lalit K. Vora, Pradeep R. Vavia, Eneko Larrañeta, Steven E.J. Bell, Ryan F. Donnelly

**Affiliations:** ^1^ School of Pharmacy Queen's University Belfast, Medical Biology Centre 97 Lisburn Road Belfast BT9 7BL UK; ^2^ School of Chemistry and Chemical Engineering, David Keir Building Queen's University Belfast Belfast BT9 5AG UK; ^3^ Department of Pharmaceutical Sciences and Technology Institute of Chemical Technology, University under Section 3 of UGC Act–1956, Elite Status and Center of Excellence–Govt. of Maharashtra Mumbai 400019 India

**Keywords:** Cholecalciferol, dissolving microneedles, Nanosuspension, transdermal

## Abstract

A nanosuspension (NS) was formulated from the lipophilic molecule cholecalciferol (CL) for enhanced transdermal delivery by embedding this NS into hydrophilic polymer‐based dissolving microneedles (DMNs). First, the NS was prepared by sonoprecpitation with different molecular weights of poly (vinyl alcohol) and poly (vinyl pyrrolidone) as stabilizers and using two different solvents for particle size and zeta potential optimization. DMN arrays were then prepared by centrifugation‐assisted micromoulding and subsequently dried. Poly (vinyl alcohol) (10 kDa) produced a NS with the lowest particle size ( ~ 300 nm). These particles yielded DMN with good mechanical properties when combined with aqueous blends of high molecular weight poly (vinyl pyrrolidone) (360 kDa). The particle size remained similar before and after MN preparation, as confirmed by scanning electron microscope. The CL was in the amorphous state in the free particles as well as in the DMN and, hence, no characteristic CL peak was observed in differential scanning calorimetry or X‐ray diffraction. DMN arrays were found to be strong enough to bear a 32 N force, showed efficient skin insertion, and penetrated down to the third layer (depth ≈ 375 μm) of the validated skin model Parafilm M®. An ex vivo porcine skin permeation study using Franz diffusion cells compared the permeation of CL from CL‐NS‐loaded DMN arrays and MN‐free CL‐NS patches. It was observed that CL‐NS‐loaded DMN arrays showed significantly higher (498.19 μg ± 89.3 μg) ex vivo skin permeation compared with MN‐free CL‐NS patches (73.2 μg ± 26.5 μg) over 24 h. This is the first time a NS of a hydrophobic drug has been successfully incorporated into dissolving MN and suggest that NS‐containing DMN systems could be a promising strategy for transdermal delivery of hydrophobic drugs.

## Introduction

Microneedle (MN) arrays have been extensively researched in the last two decades to bridge the substantial gap between transdermal patches and pain‐causing injections. MNs are minimally invasive, pain‐free, micron‐sized projection arrays (10 μm −900 μm) on baseplates capable of piercing the skin's *stratum corneum*, overcoming its barrier properties to create a transport pathway for drug molecules. MN has been shown to be suitable for self‐administration by patients (Kaushik et al., 2001; Prausnitz, 2004; Vicente‐Prez et al., 2016). A vast array of different MN types have been studied, including solid MN (Henry, McAllister, Allen, & Prausnitz, 1998), drug‐coated MN (McGrath et al., 2011), and dissolving MN (DMN). The disadvantage associated with solid MN used for pretreatment of skin is that the holes generated are quickly closed, resulting in reduced drug permeation. Drug‐coated MN can lose sharpness after coating, the loaded dose is understandably small, and sustained delivery is not achievable. Both the aforementioned MN types generate potentially biohazardous sharp waste products. On the contrary, DMNs are fabricated by using water‐soluble biodegradable polymers that completely dissolve or degrade in the skin, releasing the drug. This also eliminates any potential risk of biohazardous sharp waste. Sustained delivery from the MN baseplates is also possible for small molecules (Wu et al., 2015). DMN has been widely used to deliver hydrophilic drugs, as such molecules are easy to mix with water‐soluble polymers used for DMN preparation (Donnelly et al., 2011). Due to their insolubility in the aqueous blends typically used to cast DMN and, perhaps, a concern over solubility in skin interstitial fluid, lipophilic molecules have not typically been loaded into DMN. However, approximately 40% of new pharmaceutical drugs are which makes their efficient delivery using conventional dosage forms problematic (Dahan & Hoffman, 2008).

Dissolving microneedles efficiently deliver therapeutic agents through the dissolution of their biocompatible polymer matrices after penetration into the skin (Vora, Donnelly, et al., 2017; Vora, Sita, & Vavia, 2017). DMNs rely on the water solubility of both the loaded drug and the polymer matrix. Development of DMN for lipophilic drugs may be a challenging task, as the drug may not be uniformly dispersed in aqueous casting blends and, hence, it may be difficult to load high amounts of the drug. To facilitate solubilization of lipophilic drugs, organic solvents may be used. This can potentially lead to nonhomogeneity due to aggregation of the hydrophilic matrix‐forming polymer. The use of organic solvents can also weaken the DMN structure, as they can generate pores in the hydrophilic polymer matrix (Dangol et al., 2016).

To overcome these limitations, nanoparticles can be considered. Nanoparticles necessitate the use of high amounts of lipidic or polymeric excipients to encapsulate the drug, resulting in lower drug load in the final dosage form (Jain, Patel, Shah, Khatri, & Vora, 2016; Lai et al., 2013). The size reduction of micron‐size hydrophobic drugs to submicron size, so‐called nanosuspensions (NSs), represents an exciting colloidal delivery system (Müller & Peters, 1998). Nanosuspensions are pure forms of drug nanoparticles without any polymeric or lipidic matrix, except small amounts of stabilizer that surrounds a solid drug core. NSs, therefore, yield high drug loadings (Date & Patravale, 2004).

Particle size reduction to below 1 μm leads to increase in the surface‐to‐volume ratio of nanocrystals and an increase in aqueous solubility, as well as dissolution rate of hydrophobic molecules. NSs have been extensively explored for parenteral (Wong et al., 2008), oral (Kesisoglou, Panmai, & Wu, 2007), ocular (Kassem, Abdel Rahman, Ghorab, Ahmed, & Khalil, 2007), and pulmonary routes of application but have not been considered in detail for transdermal delivery, probably because intact particles are known not to efficiently traverse the *stratum corneum*. If pure drug nanoparticles could be deposited as a depot y DMN and rapidly dissolve in skin interstitial fluid, allowing absorption by the rich dermal microcirculation, then therapeutic plasma levels can possibly be achieved. (Donnelly & Larrañeta, 2017).

To explore this concept of NS loaded in DMN, cholecalciferol (vitamin D_3_) was used as a model lipophilic molecule. Vitamin D deficiency and its relationship with many diseases have become a major topic of medical and scientific discussion. Vitamin D is obtained from the skin's exposure to natural sunlight. Increasing vitamin D deficiency in urban populations is due to limited sun exposure (Kannan & Lim, 2013).

For oral vitamin D delivery, there are many challenges, including chemical instability and poor solubility in the gastrointestinal tract, as well as significant first‐pass metabolism, which prevents the drug from reaching the systemic circulation in its active form (Grossmann & Tangpricha, 2010). To overcome this oral bioavailability issues and to mimic the physiological condition of vitamin D synthesis in the skin, it could be delivered transdermally. To circumvent the significant barrier of the *stratum corneum*, cholecalciferol nanosuspension (CL‐NS)‐loaded DMNs were developed and characterized here. For this purpose, first CL‐NS was prepared by a sonoprecipitation method. CL‐NSs were optimized for particle size and, subsequently, loaded inside DMN by single‐step centrifugation. The developed CL‐NS‐loaded DMNs were tested for content uniformity, particle size, and ex vivo porcine skin permeation.

## Materials and Methods

### Materials

Cholecalciferol (CL, 99% purity), poly (vinyl alcohol) (PVA) (10, 31–50, and 120 kDa), poly (vinyl pyrrolidone) (PVP) (10, 40, and 360 kDa), D‐α‐tocopherol succinate (TS), acetonitrile, and methanol (both of high‐performance liquid chromatography (HPLC) grade) were all purchased from Sigma Aldrich (Poole, Dorset, UK). All other chemicals used were of analytical grade. Millipore HPLC‐grade water was used throughout the study.

### Preparation and optimization of cholecalciferol nanosuspensions

**Figure 1:**
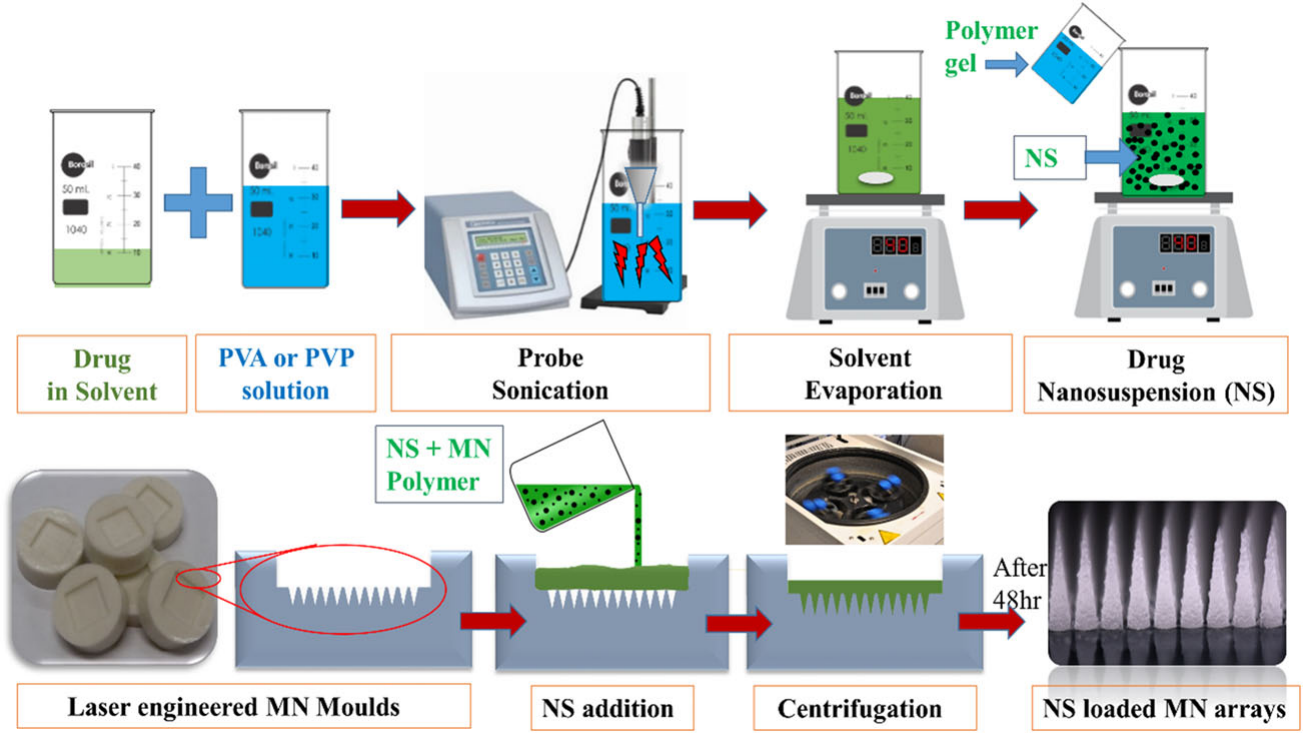
Schematic representation of fabrication of nanosuspensions and nanosuspension‐loaded microneedle arrays. PVA, poly (vinyl alcohol); PVP, poly (vinyl pyrrolidone).

Cholecalciferol nanosuspensions were prepared by a solvent evaporation antisolvent precipitation method employing probe sonication. Acetone and ethanol were selected as solvents based on the solubility of CL. CL (50 mg) and TS (20 mg) were dissolved in 1 mL of ethanol or acetone. The different molecular weights (MWs) of PVA or PVP were dissolved separately in water to prepare 2% *w*/*v* solutions. The drug solution was added dropwise over 1 min into the stabilizer solution (3 mL) under probe sonication (QS4 system, NanoLab, Waltham, MA, USA) that was continued for 5 min at an amplitude of 80% (of 125 W, 20 KHz) with 10 sec pulse on and 5 sec pulse off. The temperature of the stabilizer solution was maintained at 5°C ± 3°C during sonication. This drug dispersion was then allowed to precipitate in nanosize by solvent evaporation at ambient temperature conditions with magnetic stirring (Fig. 1).

Effect of polymer concentration, drug concentration in a solvent, the solvent to antisolvent ratio, and sonication amplitude on particle size and particle size distribution (polydispersity index, PDI) were evaluated as per Table 1. To get the powder form of optimized CL‐NS for instrumental characterization, CL‐NS were prefrozen (−70°C) for 12 h and subsequently lyophilized for 8 h with hold and ramp cycles from −40°C to 25°C.

**Table 1:** Influence of different formulation parameters on particle size and PDI value of CL‐NS.

PVA conc. (% *w*/*v*)	Solvent to antisolvent ratio	Sonication amptitude (%)	Drug conc. (mg/mL)
0.5	0.33	80	50
1	0.33	80	50
2	0.33	80	50
2	0.55	80	50
2	0.66	80	50
2	1	80	50
2	0.33	40	50
2	0.33	20	50
2	0.33	80	30
2	0.33	80	25
2	0.33	80	12.5

CL‐NS, cholecalciferol nanosuspension; PDI, polydispersity index; PVA, poly (vinyl alcohol).

### Particle size analysis and surface charge measurement

The hydrodynamic diameter (z‐average, z‐ave) of the NS was determined by dynamic light scattering, using a Zetasizer™ Nano SZ (Malvern Instruments, Worcestershire, UK). Particle size by dynamic light scattering gives the hydrodynamic radius of particles. The results are the z‐average, which is the intensity‐weighted mean diameter of the bulk population and the PDI, which is a measure of the width of the size distribution. Samples were diluted in water to a suitable concentration prior to analysis. The Zetasizer™ was also used for measurement of zeta potential. Zeta potential was measured by applying an electric field across the CL‐NS solutions using the technique of laser doppler anemometry. All measurements were carried out at 25 ± 2°C using folded capillary cells.

### Cholecalciferol nanosuspension‐loaded dissolving microneedle preparation

Dissolving microneedle arrays were prepared using the same MW polymers that were used for CL‐NS preparation. DMN arrays were prepared by centrifugation‐assisted micromoulding and subsequently dried as described previously (Donnelly et al., 2011) (Fig. 1). In detail, 3 mL of NS containing 50 mg of drug was added to 3 mL of polymeric solution, as detailed in Table 2. The homogeneity of the NS was ensured by mixing the suspension carefully for 10 min before DMN casting. Approximately 500 mg from the resulting NS‐loaded gel solution was poured into laser‐engineered poly (dimethylsiloxane) mould templates (19 × 19 and 12 × 12), centrifuged at 3500 RPM for 15 min followed by drying under ambient condition for 48 h. DMN arrays were then removed from the moulds and visually assessed for needle formation and homogeneity. To increase the loading of CL‐NS in DMN after initial optimization of polymer content, the evaporation step during CL‐NS manufacture was carried out for a longer time and at 35°C to yield approximately 30% of the initial volume and then mixed with an equal volume of 30% *w*/*v* PVP gel for further DMN preparation. DMN‐free patches (baseplates) were prepared by the same method, except using micromoulds with no projections.

**Table 2:** Formulation parameters for CL‐NS‐loaded DMN with different molecular weights of PVP and PVA.

Drug with stabilizer	Solvent (1 mL)	2% *w*/v Polymer (3 mL)	Mixing with 3‐mL polymer gel (% *w*/v) for MN
50 mg CL +20 mg TS	Acetone	PVA, 10 kDa	60% PVA. 10 kDa
		PVA, 50 kDa	40% PVA, 50 kDa
		PVA, 120 kDa	20% PVA, 120 kDa
50 mg CL +20 mg TS	Ethanol	PVP, 10 kDa	60% PVP, 10 kDa
		PVP, 40 kDa	40% PVP, 40 kDa
		PVP, 360 kDa	30% PVP, 360 kDa

CL‐NS, cholecalciferol nanosuspension; DMN, dissolving microneedle; PVA, poly (vinyl alcohol); PVP, poly (vinyl pyrrolidone); TS, tocopherol succinate.

### High‐performance liquid chromatography analysis

Analysis of CL was performed using reversed‐phase HPLC (Agilent 1200® Binary Pump, Agilent 1200®, Standard Autosampler, Agilent 1200® Variable Wavelength Detector; Agilent Technologies UK Ltd., Stockport, UK) with the detection wavelength set at 265 nm. Chromatographic separation was carried out with a Luna C (150 × 4.6 mm 18 (ODS1) column i.d. with 5 μm packing; Phenomenex, Macclesfield, UK) with 1‐mL/min isocratic elution. The mobile phase was a mixture of acetonitrile: methanol: water (90:8:2 *v*/v/v). Standard solutions of CL were prepared by diluting appropriate volumes of a stock solution of CL in methanol to give final concentrations of 0.1, 0.2, 0.5, 1, 2, 5, and 10 μg/mL. Then, 20 μL of each solution was injected onto the HPLC.

### Drug content and content uniformity

Drug content was analysed by dissolving accurately weighed CL‐NS‐loaded DMN arrays in 2‐mL water: ethanol (30:70) mixture and collecting 100 μL into 1.5‐mL tubes. This was then mixed with 0.9‐mL acetonitrile to allow precipitation of PVP polymer while the drug remained dissolved. This dispersion was centrifuged at 12,000 × *g* for 10 min, and the supernatant was collected for HPLC analysis. The content uniformity test was performed to investigate whether there was a uniform distribution of drug or drug NS within individual DMN arrays. For content uniformity within an individual DMN, percent drug recovery was determined from three different surface areas of the DMN, needles, baseplate, and the “sidewalls” formed during micromoulding (Donnelly et al., 2011) by following the same procedure as for drug content analysis. Studies were done in triplicate for each concentration. For content uniformity comparison, plain (non‐NS) CL‐loaded DMN arrays were prepared.

### Mechanical and insertion properties of cholecalciferol nanosuspension‐loaded dissolving microneedle arrays

A TA.XT‐Plus Texture Analyser (Stable Microsystem, Haslemere, UK) was used in compression mode to assess the compression and insertion properties of the NS‐loaded DMN arrays (Quinn et al., 2015). Initial heights of DMN were first determined using a stereo microscope. Then, CL‐NS‐loaded DMN arrays were attached using double‐sided adhesive tape to the movable cylindrical probe of the Texture Analyser and pressed by the test station against a flat aluminium block at a rate of 0.5 mm/sec for 30 sec and a force of 32 N (0.088 N/needle) (Eltayib et al., 2016). Pretest and posttest speeds were set at 1 mm/sec, and the trigger force was set at 0.049 N. DMN heights were measured again using the stereo microscope, and the percentage reduction in height following the application of the axial compression load was calculated. To determine insertion properties of the DMN arrays, Parafilm M® (Bemis Company Inc., Soignies, Belgium), a flexible thermoplastic sheet made of olefin‐type material was used. Parafilm M® has been previously validated as a skin simulant for insertion of MN (Larrañeta et al., 2014). The initial heights of the DMN arrays were measured microscopically prior to the test. The Parafilm M® sheet was folded into an eight‐layer film (1 mm thickness). Following attachment of the MN array to the movable probe of the Texture Analyser, the probe was lowered onto the folded Parafilm M® at a speed of 1.19 mm/sec until the required force of 32 N was exerted and held for 30 sec. The MN were then removed from the Parafilm M® sheet after insertion. The Parafilm M® sheet was unfolded, and the number of holes in each layer counted. The retrieved DMN had their heights evaluated using a Leica EZ4 D digital microscope (Leica Microsystems, Wetzlar, Germany).

### Microscopic analysis of cholecalciferol nanosuspension‐loaded dissolving microneedle arrays

The surface morphology and shape of CL‐NS‐loaded DMN arrays were examined by using a Keyence VHX‐700F Digital Microscope (Keyence, Osaka, Japan) and a TM3030 benchtop scanning electron microscope (SEM) (Hitachi, Krefeld, Germany). The latter was used in low vacuum mode at a voltage of 15 kV. In the case of NS particles, visualization inside the DMN arrays was achieved by SEM with a Quanta FEG 250 (FEI, Hillsboro, OR, USA) at an acceleration voltage of 10–20 kV under high chamber pressure (8 × 10^−5^ mbar) with standard SEM carbon tape as background.

### Differential scanning calorimetry analysis

Differential scanning calorimetry (DSC) studies of CL, TS, physical mixture, and CL‐NS‐loaded DMN arrays were carried out with a DSC Q100 (TA Instruments, Surrey, United Kingdom). Sample weights of 4.0–5.0 mg were sealed in nonhermetic‐type aluminium pans and ramped at a heating rate of 10.0°C/min in nitrogen at a flow rate of 50.0 mL/min. The DSC was calibrated with the melting temperature of indium (156.6°C).

### X‐ray diffraction measurements

The study was carried out using a benchtop X‐ray diffractometer (Miniflex™, Rigaku, Neu‐Isenburg, Germany). Radiation was from Ni‐filtered CuK, with a wavelength of 1.54 A° having a graphite monochromator. CL, TS, PVP plain polymer, CL‐NS‐loaded PVP DMN, and physical mixture of CL, TS, and PVP were respectively packed into the rotating sample holder. The obtained data were typically collected by scanning a range of 0–50°, with a scanning rate of 2°/min.

### Determination of skin penetration by optical coherence tomography

Full‐thickness neonatal porcine skin, a good model for human skin (Cilurzo, Minghetti, & Sinico, 2007; Simon & Maibach, 2000; Touitou, Meidan, & Horwitz, 1998), was used to study insertion of CL‐NS‐loaded DMN. The skin was obtained from stillborn piglets and excised within 24 h of birth using an electric dermatome (Integra Life Sciences™, NJ, USA). The skin was then wrapped in aluminium foil and stored at −20°C until use. After thawing in phosphate‐buffered saline (PBS) (pH 7.4), the skin was carefully shaved and washed with PBS before use. The skin surface was dried using tissue paper and placed dermis side down on a dental wax sheet. DMN arrays were then inserted using the Texture Analyser, with force of 32 N for 30 sec. Optical coherence tomography (OCT) images were recorded using an EX1301 OCT Microscope (Michelson Diagnostics Ltd., Kent, UK).

### Ex vivo porcine skin permeation of cholecalciferol from cholecalciferol nanosuspension‐loaded dissolving microneedle arrays

Permeation of CL‐NS‐loaded DMN arrays across dermatomed (350 μm) neonatal porcine skin was investigated ex vivo using modified Franz diffusion cells, as described previously (Quinn et al., 2015). The neonatal porcine skin was excised as aforementioned and trimmed to a thickness of 350 μm using the electric dermatome. The skin was again stored in aluminium foil at −20°C until further use. Before use, the skin was bathed in PBS to thaw and, once defrosted, carefully shaved. Sections of skin were cut by scalpel to match the diameter of the Franz cell donor compartments and carefully affixed to the donor compartment on the *stratum corneum* side using cyanoacrylate adhesive (Loctite, Dublin, Ireland), rendering the SC available for MN application. For the CL‐NS‐loaded DMN arrays (12 × 12 needle density, 600 μm height, 300 μm width at the base, and 150 μm interspacing), DMN was removed from the mould by gently squeezing the corners of the mould and sidewalls of the array were removed using a heated scalpel blade. CL‐NS‐loaded DMN arrays were then inserted using manual pressure for 30 sec applied to the MN baseplate. A cylindrical 5.0‐g stainless steel weight was placed on top of the DMN array to prevent MN expulsion, and the donor compartment of the apparatus was clamped onto the receiver compartment and sealed using Parafilm M®. The receiver compartment contained 12‐mL PBS with 2% *v*/v Tween 80 to maintain solubility of the drugs in the receiver compartment and ensure sink conditions. Syringes (1.0 mL) with long needles were used to remove 200 μL from the Franz cell contents at appropriate time points and 200 μL of prewarmed PBS was added to replace this. Samples were stored in 1.5‐mL poly (styrene) tubes. Prior to quantification of Franz cell drug content, samples were centrifuged for 5 min at 12,000 x *g* using an Eppendorf Minispin centrifuge (Eppendorf UK Ltd, Stevenage, UK). All samples were analysed using the developed reverse phase‐HPLC method. Permeation from control CL‐NS formulations was performed in the same manner, except instead of inserting a DMN array, a needle‐free patch of the same dimensions, and formulation was placed on top of the skin, followed by the stainless steel weight**.**

### Statistical analysis

The results are presented as means ± standard deviation (SD) of the mean. Statistical comparison between CL‐NS‐loaded DMN and CL‐NS films, in terms of CL permeation, was made using the Mann–Whitney U‐test by using GraphPad Prism software (ver. 6; GraphPad, Inc., San Diego, CA, USA). A difference of *p* < 0.05 was considered to be statistically significant.

## Results and Discussion

### Optimization of cholecalciferol nanosuspension

**Figure 2:**
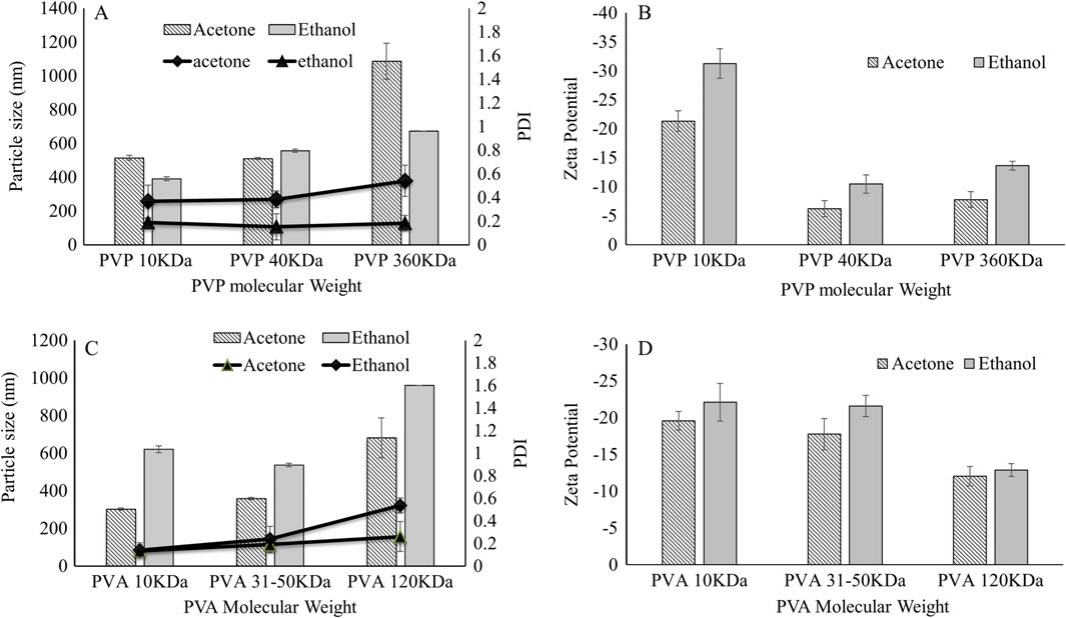
Effect of polymer molecular weight and solvent on particle size and zeta potential of NS: (A) effect of PVP molecular weight and solvent on particle size; (B) effect of PVP molecular weight and solvent on zeta potential; (C) effect of PVA molecular weight and solvent on particle size; (D) effect of PVA molecular weight and solvent on zeta potential (means ± SD, *n* = 3). PVA, poly (vinyl alcohol); PVP, poly (vinyl pyrrolidone).

For the first time, a CL‐NS was prepared by a sonoprecipitation solvent evaporation method and embedded into DMN arrays. The presence of polymeric stabilizer plays a significant role in reduction of particle size by providing a stearic adsorption barrier on the particle surface, thus retarding crystal growth or coalescence of individual particles. The ultrasonic energy created by probe sonication averts a preponderance of very large particles (Chonkar et al., 2016). Here, PVP and PVA polymers act as stabilizing agents. As MW and polymeric chain length are critical factors (Ghosh, Bose, Vippagunta, & Harmon, 2011), different MWs of both were studied to ascertain their influence on particle size and zeta potential, as shown in Figure 2. CL‐NS containing lower MW PVA and PVP exhibited lower particle size, which suggests that these lower MW polymers are able to form a uniform adsorption layer on particle surfaces, preventing coalescence of individual particles. In contrast, high MW PVA and PVP that most likely possess lower rates of adsorption on the particle surface yield larger particles. Acetone was found to yield lower particle sizes with PVA, while ethanol was found to be a more appropriate solvent for PVP. CL‐NS prepared with 10 kDa PVA produced the smallest particle size, and these particles also had the lowest zeta potentials. Because smaller particles are less likely to disrupt DMN structures following incorporation, these particles were taken forward for further DMN development.

**Figure 3:**
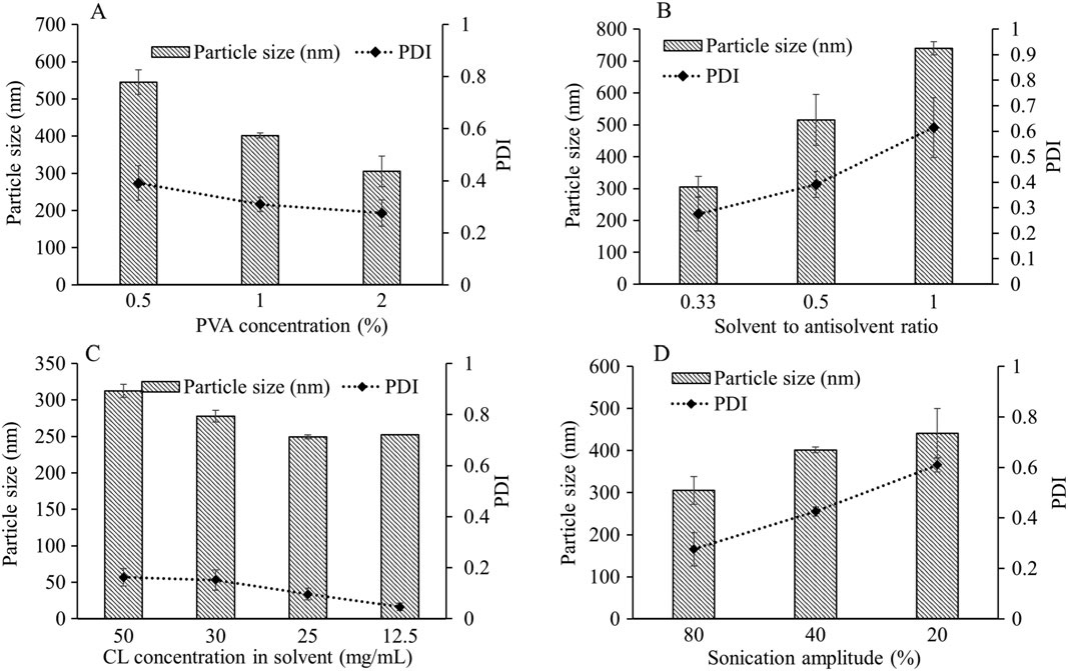
Influence of different formulation parameters on particle size and PDI value of CL‐NS: (A) effect of PVA concentration; (B) effect of solvent to antisolvent ratio; (C) effect of CL concentration in solvent; (D) effect of sonication amplitude (means ± SD, *n* = 3). CL‐NS, cholecalciferol nanosuspension; PDI, polydispersity index; PVA, poly (vinyl alcohol).

After optimization of particle size with PVA (10 kDa), the effect of formulation variables (Table 1) on particle size and PDI was evaluated, as shown in Figure 3. PVA concentration, the solvent to antisolvent ratio, and sonication amplitude all affected particle size and PDI values, while drug concentration in solvent had little effect. Increasing the amount of PVA and amplitude of probe sonication both decreased the particle size, while higher CL concentration in solvent and solvent to antisolvent ratio increased the particle size. PVA (2% *w*/*v*) with 1:3 solvent to antisolvent ratio and 80% of sonication amplitude for 5 min were found to be optimal conditions to attain mean particle size of 305.3 nm ± 32.9 nm and 0.27 ± 0.06 PDI values.

Dissolving microneedles prepared with lower MWs of PVA (10 kDa, 31–50 kDa) were found to be flexible in nature, while DMNs prepared from higher MW PVA (120 kDa) were found to be more mechanically strong. The higher MW PVA was found to be difficult to solubilize and process during DMN preparation. In the case of PVP, DMNs prepared with lower (10 kDa) and medium (40 kDa) MW PVP were broken while removing from the MN mould. DMNs prepared from higher MW (360 kDa) PVP were found to be more mechanically strong. Therefore, CL‐NS prepared using 10‐kDa PVA (302.5 nm) were mixed with higher MW PVP (30% w/v) gels for DMN array preparation.

### Characteristics of cholecalciferol nanosuspension‐loaded poly (vinyl pyrrolidone) dissolving microneedle arrays

**Figure 4:**
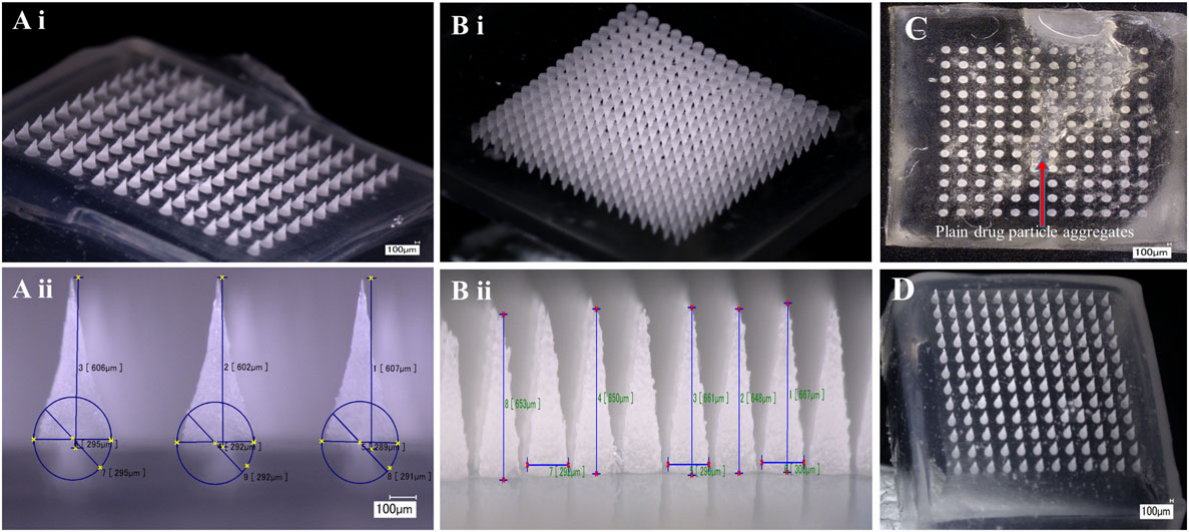
Digital microscopic images of CL‐NS‐loaded DMN arrays: (A) 12 × 12 arrays; (B) 19 × 19 DMN arrays; (C) plain CL loaded‐DMN arrays with visible aggregated drug particles; (D) CL‐NS‐loaded DMN arrays without drug aggregation. CL‐NS, cholecalciferol nanosuspension; DMN, dissolving microneedle.

The digital microscopic images (Fig. 4A and 4B) clearly showed the formation of CL‐NS‐loaded PVP DMN arrays with 12 × 12 and 19 × 19 MN arrays with sharp tips. The resulting needles measured 600 μm in height with 10% ± 1.6% *w*/w loading of CL‐NS in formulated DMN. As particle size is an extremely important stability parameter for nanoparticles, particle size was measured before (297.4 nm ± 20 nm) and after (345.7 nm ± 10.1 nm) MN preparation. The CL‐NS embedded DMN was dispersed in PBS pH 7.4 buffer without any major changes in particle size or observation of aggregation. Here, the dried state of the polymer matrix of DMN possibly acts as a stabilizer for NS particle size, preventing aggregation. The mean amount of CL recovered from CL‐NS‐loaded DMN was 94 ± 5.23%. Content uniformity within single DMN array was compared by incorporating plain drug and CL‐NS into dissolving PVP MN arrays. The percentage drug recovery with plain CL drug‐loaded DMN arrays was 179.3% ± 59%, 75.77% ± 26.6%, and 71.4% ± 16.8% and with CL‐NS‐loaded DMN arrays 98% ± 4.5%, 87.5% ± 9.7%, and 106% ± 8.21% for baseplates, sidewalls, and needles, respectively. CL particle aggregation was visible in plain CL‐loaded DMN arrays (Fig. 4C). Relatively lower values of variance for CL‐NS‐loaded DMN arrays indicate that drug nanoparticles are uniformly distributed in the entire DMN arrays (Fig. 4D). Plain CL‐loaded DMN arrays possessed varied distributions, as the micron‐sized insoluble drug particles aggregated in the water‐based polymer blends during DMN formulation. Therefore, NS is a good approach to uniformly loading lipophilic molecules in hydrophilic polymeric matrices used for DMN production.

### Scanning electron microscopy

**Figure 5:**
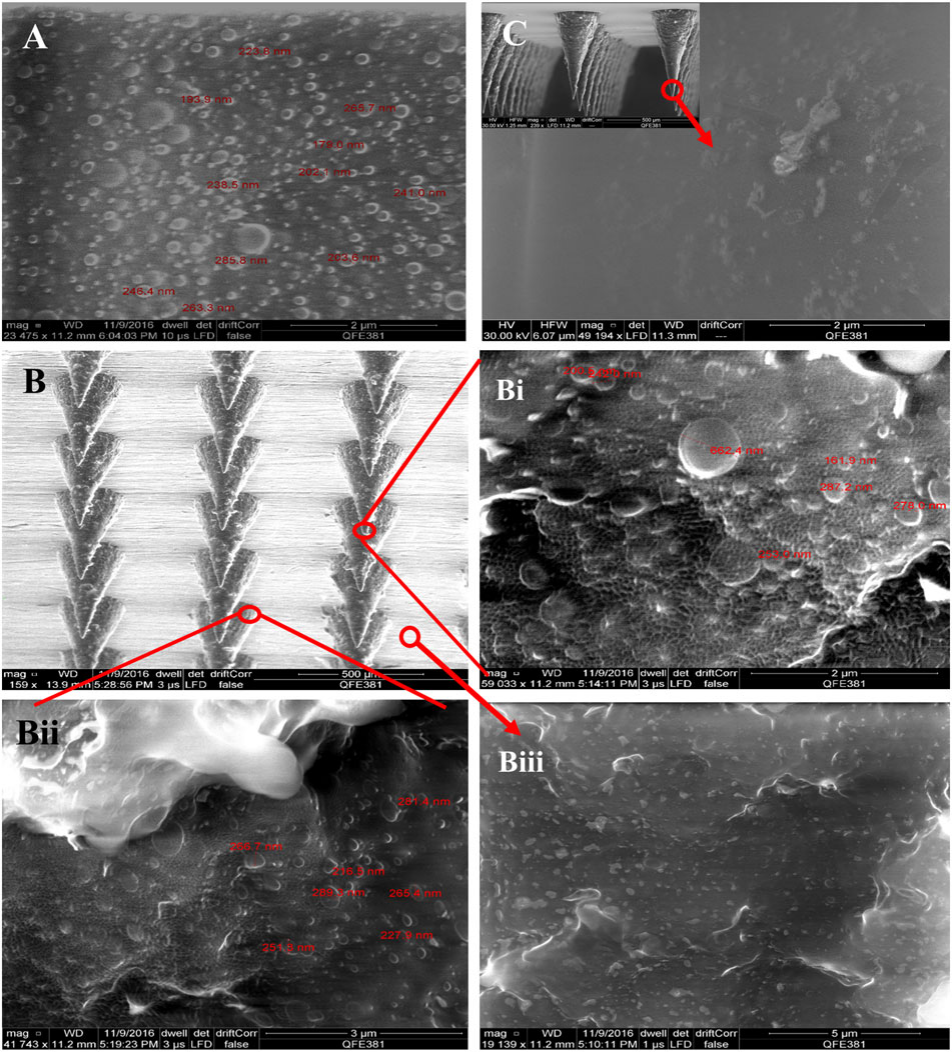
Scanning electron micrographs of: (A) CL‐NS before DMN loading; (B) CL‐NS‐loaded DMN arrays (12 × 12); (Bi) Magnified image of tips of DMN arrays; (Bii) Magnified image of a DMN array near the baseplate; (Biii) Magnified image of a DMN array baseplate; (C) plain CL drug‐loaded DMN and magnified image of its needle tips' surface. CL‐NS, cholecalciferol nanosuspension; DMN, dissolving microneedle.

Scanning electron microscope images (Fig. 5) showed spherical particle size of CL‐NS. NS embedded in DMN arrays of PVP showed a honeycomb‐like matrix with measured sizes ranging from approximately 100 nm to around 300 nm. The SEM imaging showed an even distribution of CL‐NS in the PVP DMN matrix without any evident aggregation in different parts of the DMN arrays (needle tips, near to baseplate, baseplate area). SEM images taken of plain CL drug‐loaded DMN arrays confirmed that there was no evidence of spherical particles.

### Mechanical strength and Parafilm insertion of dissolving microneedle arrays

**Figure 6:**
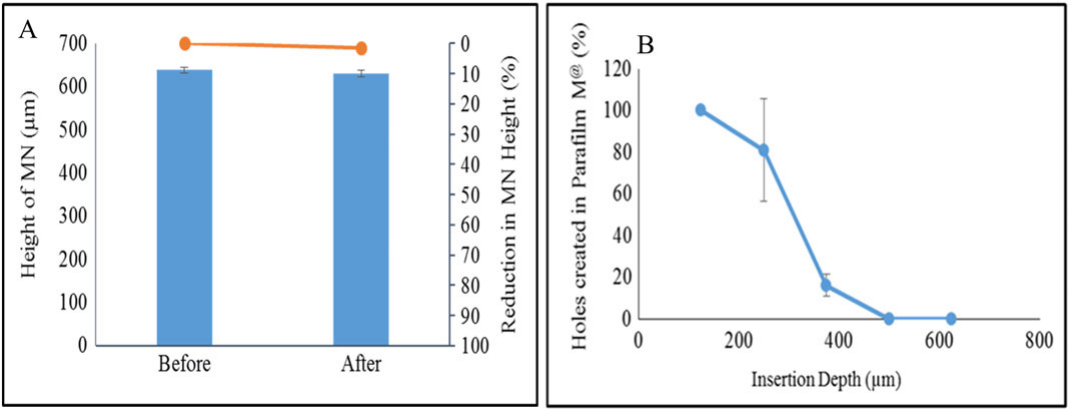
(A) Percentage reduction in height of MN upon exertion a force of 32 N at 0.5 m/sec, held for 30 sec (means ± SD, *n* = 3); (B) percentage of holes created in each Parafilm M® layer and the corresponding approximate insertion depth, using an insertion force of 32 N for CL‐NS‐loaded DMN arrays (means ± SD, *n* = 3). CL‐NS, cholecalciferol nanosuspension; DMN, dissolving microneedle.

Following application of the 32 N axial load, the CL‐NS‐loaded DMN showed a less than 2% height reduction (Fig. 6A). The CL‐NS‐loaded DMN arrays were sufficiently strong to bear the axial mechanical load of 32 N that was previously shown to be the mean force applied by human volunteers inserting DMN into their skin (Larrañeta et al., 2014). The same axial force (32 N) was also applied to evaluate the effects of insertion on needle height, using Parafilm M® as an artificial membrane to mimic the skin (Larrañeta et al., 2014). The CL‐NS‐loaded DMN arrays penetrated to the third layer of Parafilm M® (Fig. 6B). The thickness of each layer of the Parafilm M® was around 126 μm. Considering the thickness of each layer of the Parafilm M®, the total insertion depth was approximately 378 μm, which equates to more than 60% of the height of the CL‐NS‐loaded DMN arrays being inserted without any height reduction.

### Differential scanning calorimetry

**Figure 7:**
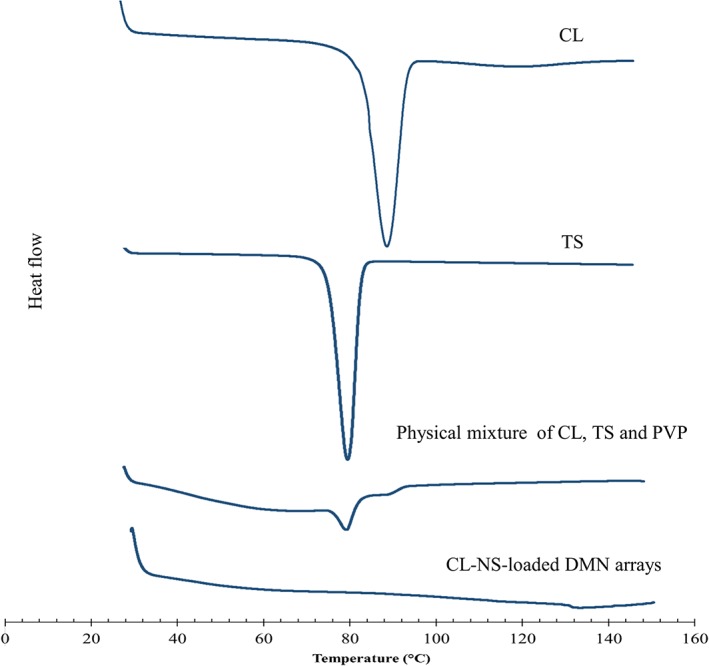
Differential scanning calorimetry thermograms of the powder samples of CL, TS, physical mixture, and CL‐NS‐loaded DMN arrays. CL‐NS, cholecalciferol nanosuspension; DMN, dissolving microneedle; TS, tocopherol succinate.

The knowledge of the crystalline and amorphous state of the drug supports understanding of the polymorphic changes that the drug may have undergone when subjected to nanosizing. Accordingly, it was deemed necessary to study whether the amorphous state was generated during the production of the NS. DSC thermograms of CL, TS, physical mixtures, and CL‐NS‐loaded DMN arrays (Fig. 7) revealed the state of CL‐NS in the DMN. DSC thermograms of pure CL and TS exhibited an intense peak at 87.9°C and 78.33°C, respectively, whereas peaks of CL embedded as NS in DMN completely disappeared. This suggests that CL in CL‐NS is in the amorphous state in the DMN.

### Powder X‐ray diffraction

**Figure 8:**
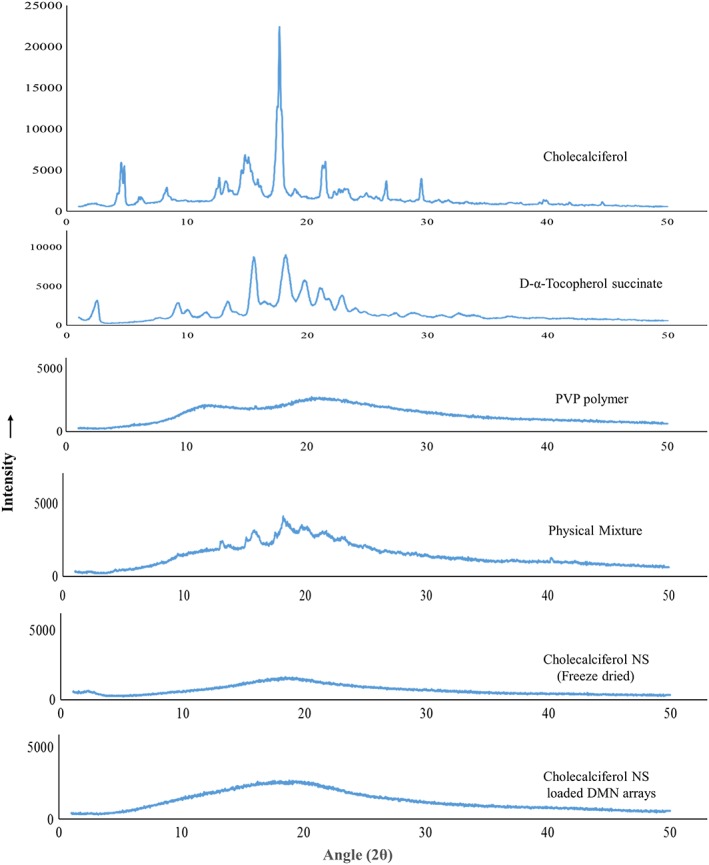
Powder X‐ray diffraction analysis pattern of CL, TS, PVP polymer and physical mixture of CL, TS and PVP, freeze‐dried CL‐NS, and CL‐NS‐loaded DMN arrays. CL‐NS, cholecalciferol nanosuspension; DMN, dissolving microneedle; PVP, poly (vinyl pyrrolidone); TS, tocopherol succinate.

Powder X‐ray diffraction studies are used to understand the nature of core material in polymeric matrices. Powder X‐ray diffraction patterns (Fig. 8) of CL exhibited characteristic crystalline peaks at a 2θ angle of 4.56, 4.8, 13.24, 14.58, 14.86, 17.72, 21.54, 22.64, 26.62, and 29.6, respectively, indicating its highly crystalline nature. In contrast, the characteristic drug peaks were found to be considerably lowered when CL NS was loaded into DMN, further suggesting that CL was present in an amorphous state.

### Determination of skin penetration

**Figure 9:**
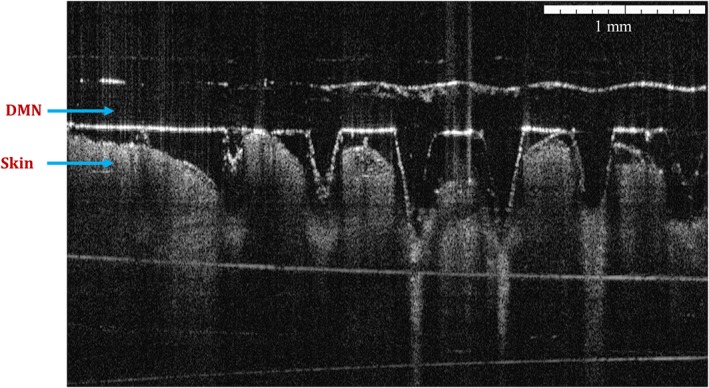
Optical coherence tomography images after insertion of CL‐NS loaded DMN arrays into the excised full thickness neonatal porcine skin in vitro. CL‐NS, cholecalciferol nanosuspension; DMN, dissolving microneedle.

A non‐invasive optical imaging technique, OCT, was used to acquire real‐time images of the insertion of these CL‐NS‐loaded DMN arrays into the neonatal porcine skin (Fig. 9). These results indicated that DMN arrays possessed the capability to be inserted into the neonatal porcine skin, reaching insertion depths of approximately 350–400 μm. The skin insertion profiles obtained by OCT are consistent with the insertion depths obtained with Parafilm M® layers. Thus, more than 60% of the MN height was actually residing within the skin and, therefore, this was the portion of the DMN available for in situ dissolution and release of the nanoparticulate drug intradermally.

### Ex vivo porcine skin permeation of cholecalciferol from cholecalciferol nanosuspension‐loaded dissolving microneedle arrays

**Figure 10:**
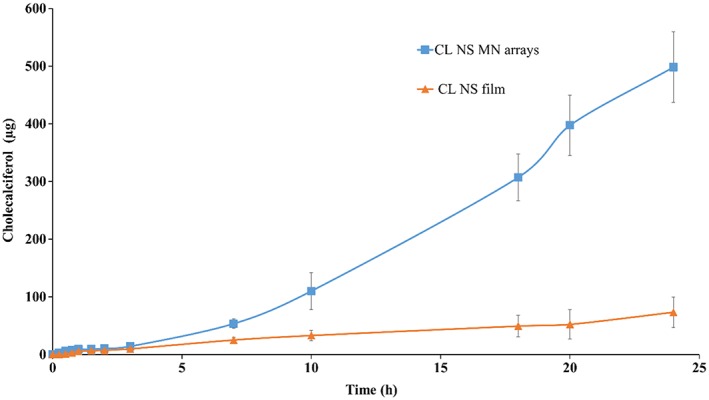
Ex vivo permeation profile of CL across dermatomed (350 μm) neonatal porcine skin over 24 h when delivered using CL‐NS‐loaded DMN arrays in comparison with CL‐NS‐loaded MN free films (means ± SD, *n* = 3). CL‐NS, cholecalciferol nanosuspension; DMN, dissolving microneedle.

The ex vivo permeation profiles of CL across dermatomed (350 μm) neonatal porcine skin over 24 h when delivered using CL‐NS‐loaded DMN arrays and CL‐NS‐loaded DMN‐free films are shown in Figure 10. Permeation of CL across neonatal porcine skin was significantly enhanced by using CL‐NS‐loaded DMN arrays in comparison with the CL‐NS‐loaded DMN‐free films (*p* < 0.05). Following application of the DMN for 24 h, 498.19 μg ± 89.3 μg CL was delivered from CL‐NS‐loaded DMN arrays, in comparison with 73.2 μg ± 26.51 μg delivered from the film containing a CL‐NS without DMN. Therefore, the results demonstrate that the CL‐NS‐loaded DMN arrays significantly assist the delivery of the CL through the skin to achieve enhanced transdermal delivery. It is likely that nanoformulation enhances CL solubility in aqueous fluid relative to the normal micronized drug particles and that the DMNs allow delivery of the nanosized drug by permeating the *stratum corneum* barrier. Thereafter, the deposited NS dissolves in skin interstitial fluid allowing systemic drug absorption.

## Conclusion

This is the first time a NS of a hydrophobic drug was successfully optimized and incorporated into DMN arrays. The DMN forming polymer may have acted as a stabilizer for particle size after DMN preparation. NS particles were uniformly distributed in nanosize without any aggregation due to the spatial separation of particles within the polymeric matrix of DMN arrays. This NS formulation may be able to deliver a wide range of lipophilic compounds intradermally when combined with DMN. Such a novel NS‐loaded MN system might be helpful in placing the nanodrug depots into the skin for enhanced transdermal delivery. Once in the skin, the solubility‐enhancing properties of nanoformulation should enable drug dissolution in skin interstitial fluid for subsequent absorption by the rich dermal microcirculation. In conclusion, this proof‐of‐concept work, therefore, represents a significant progression in the utilization of MN technologies in combination with drug NSs for delivery of lipophilic drugs into the viable skin (epidermis and dermis) layers for maximum therapeutic gain and patient benefit.

## Conflict of Interest

None declared.
